# Cost-effectiveness analysis of a postoperative 48-hour care bundle for high-risk patients undergoing abdominal surgery

**DOI:** 10.1371/journal.pone.0320968

**Published:** 2025-06-03

**Authors:** Cleiton Pando, Ana Paula Etges, Miriam Z. Marcolino, Ricardo B. Cardoso, Adriene Stahlschmidt, Carise A. Polanczyk, Luciana C. Stefani

**Affiliations:** 1 Programa de Pós-Graduação em Ciências Médicas, Faculdade de Medicina, Universidade Federal do Rio Grande do Sul (UFRGS), Porto Alegre, Brazil; 2 Serviço de Anestesia e Medicina Perioperatória, Hospital de Clínicas de Porto Alegre (HCPA), Porto Alegre, Brazil; 3 Departamento de Epidemiologia, Universidade Federal do Rio Grande do Sul (UFRGS), Porto Alegre, Brazil; 4 National Institute of Science and Technology for Health Technology Assessment, INCT/IATS, Porto Alegre, Brazil; 5 Avant-garde Health, Boston, Massachusetts, United States of America; 6 Departamento de Cirurgia, Faculdade de Medicina, Universidade Federal do Rio Grande do Sul (UFRGS), Porto Alegre, Brazil; Sengkang General Hospital, SINGAPORE

## Abstract

**Background:**

Enhancing surgical care pathways, including scaling-up care after surgery, seeks to deliver better patient-centered care and improve outcomes. However, the value associated with these pathways need to be investigated.

**Methods:**

We analyzed the potential increase in efficiency of a new care pathway, a postoperative 48-hour care bundle designed to enhance care for high-risk patients undergoing major abdominal surgery, based on micro-costing and cost-effectiveness analyses. Data from a prospective cohort of patients who underwent a high-risk surgical bundle were compared with those who received the standard care regimen. The bundle included standardized risk communication using the ex-care model, implementing a high-risk patient discharge checklist from the recovery room to the ward, ensuring prompt nursing admission to the ward, increasing the frequency of vital sign monitoring in the surgical ward, conducting troponin measurements, and providing rapid access to medical support when needed. Costs were calculated individually throughout the postoperative period until discharge following the time-driven activity-based costing method. One-year survival rates were evaluated using Kaplan-Meier survival curves. A cost-effectiveness analysis was conducted in terms of life-year gain (LYG) and incremental cost-effectiveness ratio (ICER).

**Results:**

Data from 130 and 188 patients in the bundled and non-bundled groups, respectively, were evaluated. The mean cost per patient of the high-risk surgical bundle was US$2114.63 while for the conventional care group it was US$1447.86. The Kaplan-Meier curve for one-year mortality showed that the groups differed significantly (p =  0.002). According to the model, the new care strategy incremented 0.12 life-year gained at an incremental cost of $150.12. The projected incremental cost-effectiveness ratio for one year of life was $1217.66.

**Conclusion:**

Our results suggest that implementing a high-risk surgical bundle can enhance healthcare delivery efficiency, making it a valuable and cost-effective strategy for high-level testing to create a more sustainable healthcare system.

## Introduction

Providing access to timely and safe surgical care is an essential component of any health care system. In low- and middle-income countries (LMIC), an increase in complications and deaths after surgery in high-risk patients is anticipated, mainly because of the inability to rescue patients after they develop common postoperative complications [1,2]. In these settings, surgical mortality can be twice the global average [3]. Strategies focused on improving the quality of postoperative care have been investigated in several countries with the intention of implementing more effective surgical services [4–6]. However, to translate these positive outcomes into a strategy that policymakers can adopt as an efficient way to structure surgical services, a more detailed understanding of patient-level costs and sequential effectiveness is required.

Cost-effectiveness analysis (CEA) is most often associated with the evaluation of therapies, medications, or devices, in which spending information is largely concentrated on the costs of medical technology. When evaluating redesigned services, a more granular understanding of service costs is required, which can be achieved through micro-costing studies [7]. Analyzing costs at the patient level allows for an accurate assessment of the impact of a redesigned service in healthcare, enabling the identification of reference care strategies [8]. There is a notable lack of data in the surgical field regarding the evaluation of perioperative redesign projects, particularly in terms of assessing critical clinical and economic outcomes and the resources required [9].

The time-driven activity-based costing (TDABC) method [10,11] is an effective technique for measuring healthcare costs. This enables providers to reduce waste without compromising outcomes and, in some cases, even improve them [12]. Applying the TDABC method to surgical pathways provides a comprehensive overview of costs throughout the care cycle, offering detailed information on the surgical process and supply use [13]. This detail in cost-effectiveness analysis (CEA) ensures that the results accurately represent the efficiency of a redesigned service [9].

We recently published the clinical results of implementing a surgical pathway in a public teaching hospital in Brazil to improve postoperative care processes [14]. This pathway encompassed a series of standardized measures aimed at improving postoperative care for high- and very-high-risk patients identified by the Ex-Care risk model [21]. It included standardized risk communication, to ensure proper transfer of patients from the recovery room to the surgical ward, and a discharge checklist addressing fluid balance, laboratory results, and prescription reviews. Nurse handovers from the Post-Anesthesia Care Unit (PACU) to the surgical ward were enhanced, ensuring prompt ward admission within 30 min of PACU discharge and comprehensive information sharing among caregivers. The high-risk surgical bundle was prescribed for 48 h and involved an increased frequency of vital sign monitoring, with checks every three hours. High-sensitivity troponin T measurements were collected preoperatively and at 24 and 48 h postoperatively to monitor for myocardial injury, with cardiology evaluations triggered if levels exceeded 65 ng/mL or if an absolute change greater than 40 ng/mL between pre- and postoperative peaks was detected. The pathway also included multidisciplinary care and rapid support mechanisms. Daily ward rounds were supervised by a consultant anesthetist and internal medicine teams were involved in cases of clinical deterioration, whereas senior surgeons and their teams intensified postoperative care supervision. This structured pathway ensured comprehensive and timely care, prioritizing early detection and management of complications to improve patient outcomes.

This strategy has already demonstrated a positive clinical impact by increasing the identification of clinical deterioration and scaling-up care, which reduced the in-hospital death risk from 10.5% to 6.3%, with relative risk (RR) 0.46 (95% CI: 0.3–0.72) [14].

Our aim was to measure the potential cost-efficiency increase at the system level of implementing a postoperative pathway bundle for high-risk surgical patients undergoing major abdominal surgery, based on micro-costing and cost-effectiveness analysis. We sought to determine whether this intervention had the potential for widespread adoption in low-resource settings.

## Materials and methods

This was a secondary planned cost analysis from a quality improvement program designed to improve outcomes in a cohort comprising 1189 high-risk surgical patients at a public, high-complexity hospital in Brazil [14]. All patients who underwent major abdominal surgeries were included in the analysis (see S1 Box in S1 File). Those assigned to the new high-risk surgical bundle were considered from the 2019 and 2020 period, while those in the usual care group were included from 2015 to 2016. Data were obtained through a retrospective review of medical records conducted between September 1, 2020, and May 31, 2022.

We used a mixed approach combining cost-effectiveness and micro-costing analyses [15]. For CEA, the population of interest was high-risk patients who underwent abdominal surgery. Within this group, we selected patients who underwent colon surgery for the microcosting analysis using the TDABC method. All patients who underwent this procedure during the prospective and retrospective periods were included. We chose colon surgery due to its high frequency and lower variability in care, which enabled the uniform and detailed granular data collection required for this sub-analysis.

We compared the earlier period of costs (September 2015 to December 2016) with those during the implementation of the high-risk surgical bundle (January 2019 to January 2020). The results were used to evaluate the cost-effectiveness of the redesigned enhanced perioperative services.

Formal ethical approval to analyze the anonymized patient data was obtained (CAAE 3266891900000532). Given that this study was a retrospective analysis, informed consent from patients was waived, and the retrospective data were processed anonymously to ensure privacy and confidentiality. However, for the micro-costing analysis using the TDABC methodology, which involved calculating the average time spent by each healthcare professional in patient care, written informed consent was obtained from the healthcare professionals involved.

### Micro-costing analysis following the TDABC method

**Fig 1:**
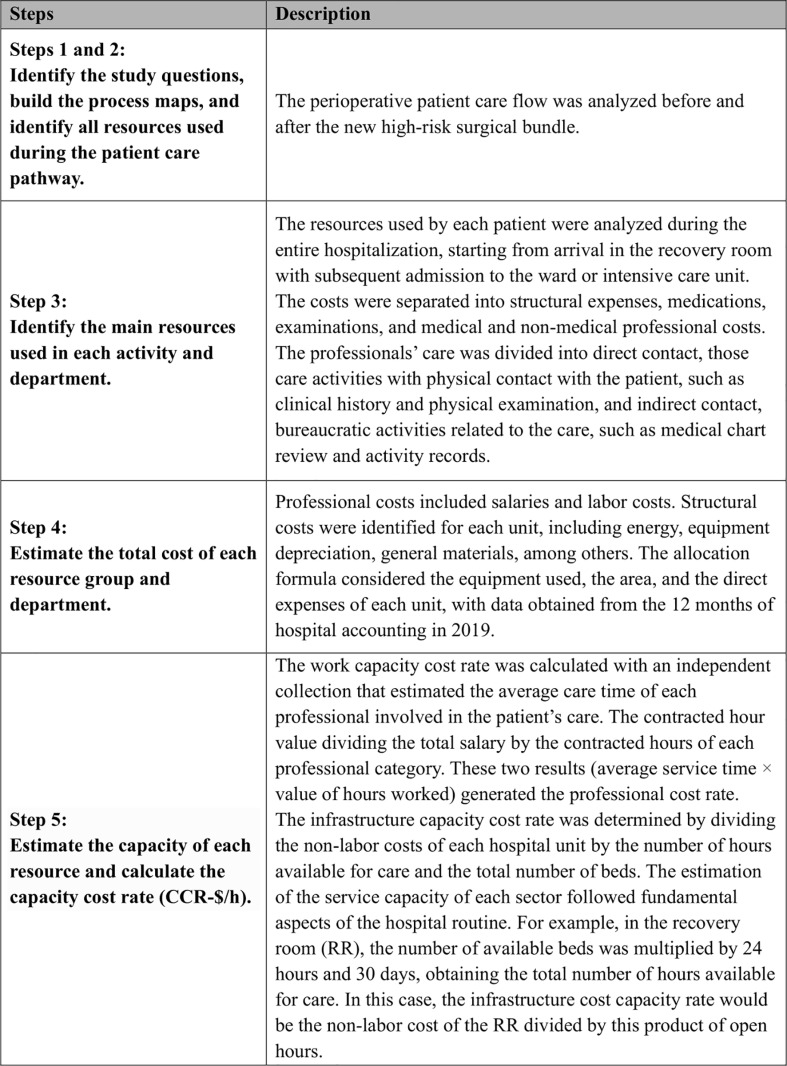
Summary of the methodology. Summary of the methodology applied for the use of the micro-costing technique – time-driven activity-based costing method (TDABC), based on 8 steps.

A micro-costing, individual patient-level TDABC method was used to estimate real-world resources from patients [10,16]. All resources consumed in activities related to the healthcare process during the postoperative period until discharge were identified, such as structural expenses (such as depreciation, energy, general material, taxes), examinations, medication, and professional services. Fig 1 shows the steps used to determine the TDABC analysis [12]. All costs were discounted at the 5% level. Costs in the local currency (Brazilian Reais – R$) were converted into American dollar ($) according to the October 2022 exchange rate. The costs obtained by the micro-costing analysis were extended to all patients for CEA.

Costs were also analyzed in subgroups of the three most frequently observed complications: hypotension (defined as a symptomatic drop in blood pressure that required treatment – crystalloid push or vasoactive drug), infection (defined as antibiotic use up to the seventh postoperative day), and acute kidney injury (AKI) (increase of 1.5 the baseline value creatinine) [17].

### Effectiveness measure

One-year survival was evaluated using Kaplan–Meier survival curves comparing the groups using a log-rank test to capture the clinical benefit of the strategy. Data on mortality within 30 days and one year were obtained by consulting the hospital’s database or telephone. Patients still alive were censored at one year of follow-up. A Cox proportional hazards model, with the intervention group as the exposure (high-risk bundle), adjusted to the antibiotics use during the postoperative period, the risk class based on the Ex-Care risk model, which included clinical (ASA physical status and age) and the surgical (nature of surgery and severity) factors, was used to analyze the hazard ratio for the primary outcome (one-year mortality).

Cost-effectiveness analysis was carried out to compare both pathways (the standard of postoperative care for high-risk patients and the new bundle of care) in terms of life-year gain (LYG) and incremental cost-effectiveness ratio (ICER). We calculated the difference in total hospital costs and the difference in primary outcome (one-year mortality) between the two cohorts, with the results shown as an ICER.

A deterministic sensitivity analysis was performed to identify the variables that could significantly influence the final results. All analyses were repeated considering the presence or absence of infection given the high incidence of this complication. The ICER evaluation followed the national regulations to guide the technology incorporation process. In December 2022, the Brazilian Ministry of Health published a recommendation put forward by the National Committee for Health Technology Incorporation in the Unified Health System (CONITEC), which stipulated that an ICER not exceed the gross domestic product (GDP) per capita as an effective limit of approximately $7732.46 [18].

### Statistical methods

Continuous variables were presented as medians and interquartile ranges, and categorical data were reported as frequencies and constant variables. Sample and micro-costing data were consolidated in a Microsoft Excel spreadsheet for Mac 2019. Proportional hazards assumption was analyzed using Shoenfeld residuals, including global and individual covariate testing. A p < 0.05 was considered statistically significant. Survival curves, regression, and sensitivity analyses were performed using R version 3.4.3 [19]. The results were reported according to the Consolidated Health Economic Evaluation Reporting Standards (CHEERS) checklist [20].

## Results

**Fig 2:**
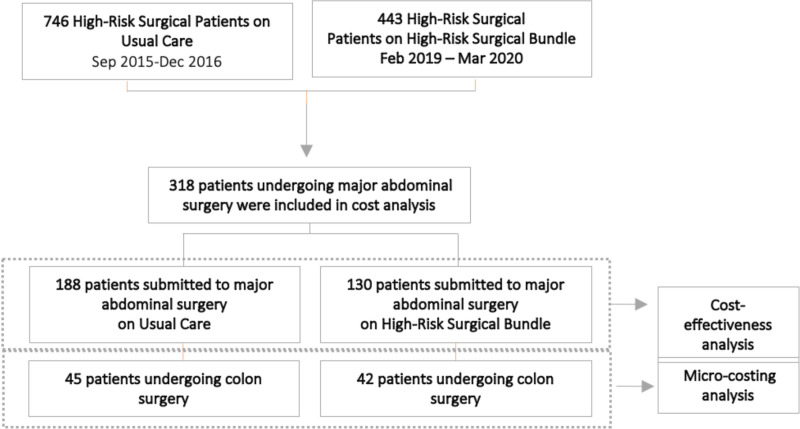
Study flow. The study flow is demonstrated with the allocation of patients into two groups (new bundle and usual care). Additionally, the identification is carried out to determine which patients underwent cost analysis through micro-costing and which patients underwent cost-effectiveness analysis.

Our study included 318 patients undergoing major abdominal surgery, of whom 130 were allocated to the high-risk bundle group and the remaining 188 to the usual care group. A total of five patients were excluded from the one-year analysis (two patients from the new line of care and three patients from the usual care) due to loss to follow-up. Fig 2 illustrates the flow of this study.

The mean age of the patients in the high-risk bundle was 67.7(SD =  10.06), 65 (50%) were men, and 125 (96.15%) were classified as ASA class 3. The mean age of the usual care patients was 66.1 years (SD =  12.30); 103 (54.78%) were men, and 159 (84.57%) were ASA 3. Patients in both groups had a baseline risk of death of > 5% in 30 days, according to the Ex-Care risk model [21]. The proportion of very high-risk patients (≥10% 30-day risk of death) was 48.46% (63/130) in the high-risk surgical bundle group, and 44.15% (83/188) in the usual care group. Urgent surgical procedures were performed in 29% and 46.8% of the high-risk surgical bundle and usual care groups, respectively. The most frequent abdominal surgeries were colon surgery and exploratory laparotomies. The postsurgical infection rate was 38.5% in the intervention group and 27.1% in the control group. For up to 30 days, surgical reinterventions occurred in 32/130 patients in the intervention group (24.6%) and in 11/188 patients in the control group (5.8%). The mean length of hospital stay was 19 (SD =  22.21) days in the intervention group and 16 (SD =  11.64) days in the usual care group. The occurrence of death within 30 days and one year was 3.85% (5/130) and 16.15% (29/188), respectively, in the high-risk bundle group and 15.42% (21/130) and 31.91% (60/188), respectively, in the usual care group. Table 1 shows the main characteristics and findings for each group.

**Table 1:** High-risk patient characteristics of those receiving enhanced or usual care.

Characteristics	High-risk bundle *n* = 130 patients	Usual care *n* = 188 patients	*p*-value
Demographic			
Age (years)	67.7 (SD = 10.06)	66.1 (SD = 12.30)	0.241^a^
Male	65 (50%)	103 (54.78%)	0.401^b^
**Composite risk scales**			
ASA-OS			0.008^c^
II	2 (1.54%)	13 (6.91%)	
III	125 (96.15%)	159 (84.57%)	
IV	3 (2.31%)	12 (6.38%)	
V	0	4 (2.13%)	
EX-CARE			0.448^b^
Risk between 5% and 10%	67 (51.54%)	105 (55.85%)	
Risk > 10%	63 (48.46%)	83 (44.15%)	
**Operative**			
Procedure type			0.053^b^
General	14 (10.77%)	39 (20.74%)	
Digestive surgery	66 (50.77%)	75 (39.89%)	
Proctology	34 (26.15%)	43 (22.87%)	
Gynecology	7 (5.38%)	8 (4.6%)	
Urology	9 (6.93%)	23 (12.24%)	
Nature			<0.001^b^
Elective	92 (70.77%)	100 (53.19%)	
Urgency/Emergency	38 (29.23%)	88 (46.81%)	
Laparoscopic Surgery	20 (15.3%)	46 (24.4%)	
Surgical time (min)	170 (SD = 82.84)	150 (SD = 83.58)	0.043^a^
Postoperative infection	50 (38.46%)	51 (27.12%)	0.033^b^
Reintervention (30 days)	32 (24.61%)	11 (5.85%)	<0.001^b^
Hospitalization time (days)	19 (SD = 22.21)	16 (SD = 11.64)	0.334^d^
Death (30 days)	5 (3.85%)	29 (15.42%)	0.002^b^
Death (1 year)	21 (16.15%)	60 (31.91%)	0.002^b^

^a^Independent samples *t*-test.

^b^Pearson’s Chi-squared test.

^c^Pearson’s chi-squared test with a simulated p-value (based on 10 000 replicates).

^d^Kruskal–Wallis test.

SD =  standard deviation.

### Micro-costing analytics

A detailed micro-costing analysis was performed in a subgroup of 87 patients undergoing colon surgery, 42 patients in the new high-risk surgical bundle, and 45 patients undergoing usual care.

**Fig 3:**
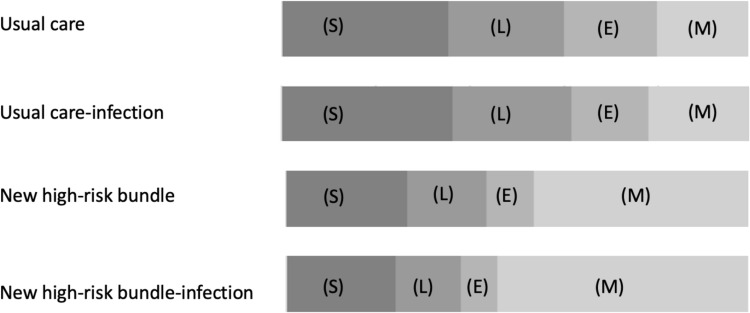
Cost composition of patients: new bundle versus usual care. The chart illustrates the differences found in the cost composition of patients undergoing the new bundle (n = 42) versus usual care (n = 45). We can also observe the distinctions in cost composition among patients who developed an infection. (New bundle n = 15 and usual care n = 18). S =  structural costs; L =  labor costs; E =  examination costs; M =  medication costs.

The mean cost per patient in the high-risk bundle group was $2114.63, whereas in the usual care group it was $1.447.85, which constituted a 46% higher expense in the intervention group. The increment in costs in the high-risk bundle group was mainly attributed to medication and medical labor costs. In the intervention group, the medication expenses were $985.69, whereas in the usual care group they were $282.33. Labor costs were 23% higher in the high-risk bundle group, whereas medication was 249% higher, suggesting that although the surgical redesign initiative changed the way in which labor costs deliver care, the incremental cost was mainly attributed to medication consumption. Fig 3 shows the composition of costs, and Table 2 and S1 Table in S1 File outline the detailed values for each cost category.

**Table 2:** Differences in mean costs and cost composition between patients undergoing the new bundled care approach and those receiving usual care. The costs associated with patients who developed infections in both groups were also described.

	New high-risk surgical bundle			Usual care		
Cost $ Mean (SD)	**All patients**	**Presence of Infection**		**All patients**	**Presence of Infection**	
	*n* = 42	Yes (n = 15)	No (n = 27)	*n* = 45	Yes (n = 18)	No (n = 27)
Total	2114.63 (3707.60)	4046.92 (5759.62)	1041.13 (668.00)	1447.86 (1067.81)	1999.46 (1182.43)	933.05 (690.94)
Medication	985.69 (3233.51)	2196.22 (5285.10)	313,17 (356.73)	282.33 (561.64)	440.25 (818.85)	152.93 (218.80)
Structure	554.21 (617.73)	960.05 (854.97)	328.75 (241.03)	518.45 (376.24)	724.87 (354.92)	328.98 (278.63)
Examinations	215.66 (249.23)	316.44 (364.08)	159.67 (129.66)	287.00 (282.28)	328.48 (247.09)	224.03 (258.43)
Other professionals Direct contact	86.68 (80.34)	136.50 (111.08)	59.00 (35.48)	99.65 (55.15)	131.78 (58.85)	67.59 (34.87)
Other professionals Indirect contact	142.71 (125.17)	219.76 (170.64)	99.91 (59.69)	160.80 (95.94)	218.88 (110.88)	105.47 (51.19)
Medical Direct contact	56.76 (64.34)	94.87 (87.12)	35.59 (31.10)	46.05 (51.70)	72.78 (62.19)	24.38 (28.99)
Medical Indirect contact	72.91 (86.18)	95.47 (115.02)	45.04 (47.37)	53.57 (65.66)	82.41 (74.60)	29.67 (44.03)

$ =  US dollar.

SD =  standard deviation.

### Subgroup analysis

Index complications were identified as the first markers of clinical deterioration. The most prevalent complications were hypotension, present in 40% of patients in the high-risk bundle group and in 44% of the usual care group. The cost of patients with hypotension increased by 25%. Infection was present in 36% (15/42) of the patients in the new care bundle, resulting in the greatest difference in the mean cost, with an increase of 91%. Infection was also common in usual care (40%, 18/45), which generated an average increase in the total cost of 38%. Acute kidney injury (AKI) occurred in 26% (11/42) of the high-risk bundle group and in 20% (9/45) of the usual care group, which increased the average total cost by 68% and 59%, respectively. See S1 Figure in S1 File for further details.

Due to the considerable cost increase for patients who presented with an infectious condition in both groups, CEA was also stratified between patients with or without an infection. Tables 1 and 2 show the means and SD of these groups. The mean cost of patients in the high-risk bundle group with an infectious condition in the postoperative period was $4046.92, which increased the mean cost of these patients by 102% compared with the usual care group with an average cost of $1999.00. Medication consumption mainly justifies these incremental costs, registering a 399% higher cost in the intervention group, followed by indirect contact with physicians (49%), structural expenses (32%), and medical labor charges per direct contact (30%).

### Cost-effectiveness analysis

CEA was performed for all high-risk patients who underwent abdominal surgery. CEA was constructed using a reference pathway (usual care) for high-risk patients and a new care bundle. Data on mortality within 30 days and one year were obtained by consulting the hospital’s database or telephone. The Kaplan-Meier curve for one-year mortality (S2 Figure in S1 File) shows that the groups were significantly different (p =  0.002). S2 Table in S1 File shows the details of the median comparisons used for the ICER.

To identify the main confounders for the one-year survival benefit, we carried out Cox regression analysis, which confirmed the protection of the high-risk bundle (hazard ratio (HR) 0.40 (95% CI: 0.24–0.66; p =  0.0003). The risk class greater than 10% of the probability of death had an HR of 2.29 (95% CI: 1.42–3.67; p =  0.0005), and the presence of postoperative infection had an HR of 1.86 (95% CI: 1.17–2.93; p =  0.007) (S3 Table in S1 File).

**Fig 4:**
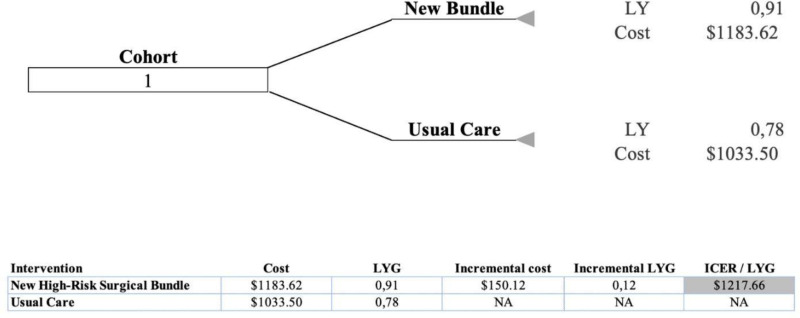
Decision tree showing cost-effectiveness analysis. This decision tree illustrates the increase in patient survival who underwent care under the new bundle and the economic impact of this novel care approach. LY =  life year; LYG =  life year gained; ICER =  incremental cost-effectiveness ratio; $ =  US dollar.

According to the model, the base scenario with the decision tree over a life year showed that using the new care strategy resulted in an increment of 0.12 LYG at an incremental cost of $150.12. The projected ICER for the one LYG was $1217.66, as shown in Fig 4.

A scenario was also constructed with the presence of infection in both groups; in this case, again, the increase in LYG was 0.12 for the intervention group, generating an additional cost of $257.35, generating an ICER for one LYG of $2203.32 (S3–S5 Figures in S1 File).

## Discussion

We demonstrated that our redesigned pathway for improving postoperative care in high-risk surgical patients effectively enhanced system sustainability. A micro-costing analysis using the TDABC method showed that the incremental cost of the high-risk surgical bundle is justified by a significant reduction in one-year mortality, with an ICER of $1217.66 per life year gained. The granularity of the micro-costing analysis also reveals potential cost-saving opportunities, further increasing the efficiency of the strategy.

The perioperative period is a complex system [21] compounded by several layers of integration between health access, structure, and processes encompassing the quality of care provided [8,22,23]. The TDABC method is an emerging cost analysis method that contributes to increasing the accuracy of cost evaluations and designing effective solutions. Its use made it possible to obtain the resources spent at each perioperative step regarding structure, medication, examinations, and professional costs [12] and allowed for a confident estimation of the financial impact of the new high-risk pathway. The observed mild cost increase for high-risk patients undergoing the new pathway is mainly attributed to medication expenses. We observed a clear escalation of care triggered by the shorter interval for measuring vital signs and the intensification of clinical teams’ assistance [14], with consequent escalation of medication use, such as antibiotics. Interestingly, despite introducing the biomarker troponin in the new high-risk pathway, laboratory expenses did not differ among the groups. This is of utmost importance because cost is an obstacle to the routine use of troponin dosage in constrained resource scenarios. Therefore, at least in high-risk patients, our results suggest that troponin dosage is not associated with increased perioperative costs.

Our micro-cost analysis also demonstrated that postoperative complications such as infection, hypotension, and acute kidney injury were associated with increased costs in both the intervention and usual care groups. Infection was the leading cause of increased expenses, with a 102% increase in the intervention group’s average cost.

High-risk surgical patients are responsible for a significant number of complications and deaths [13,24,25]. Pathways designed to improve the perioperative processes for vulnerable high-risk patients have been implemented in different contexts, such as the ASOS-2 study in Africa, in which there was a proposal for improved postoperative surveillance, and the EPOCH study, which analyzed interventions to improve survival in emergency laparotomies [26,27]. These studies demonstrated variable clinical results and incipient economic evaluation [26,27]. In a recent systematic review of perioperative care pathways in LMICs [28], the cost of interventions and lack of available resources were identified as the main barriers to implementing care pathways. Indeed, economic evaluations of enhanced recovery after surgery programs are evolving, and the reduction in fragmentation of care and wasteful resource utilization have been heralded as sustainable solutions [9,29]. A multidimensional evaluation of enhanced recovery after joint arthroplasty surgery [30] showed that resources invested in training activities are largely depreciated by benefits once technology is permanently introduced.

Implementing value-based perioperative care is challenging; however, there is a growing consensus that this is a way to increase the sustainability of healthcare services [31]. The development of studies based on accurate financial information about the processes to deliver care represents an important contribution to guiding more effective and sustainable health policies, which have enormous potential to increase the value of healthcare.

The main strength of this study is the micro-costing analysis, which showed the potential benefit of micro-costing analysis in a surgical design pathway. However, this study had some limitations. First, it was a secondary analysis of a before-and-after study, and data from the usual care group were retrospectively collected. Due to the non-randomized nature of the study, differences between groups in surgical variables were observed; however, we applied a statistical approach to control for these differences in all analyses. Second, this was a unicentric economic evaluation conducted at a hospital in the Brazil’s public health system, which requires external validation. Third, micro-costing analysis was performed in a subsample, and the values were extrapolated for the entire sample in the CEA. This may have affected the ICER; however, we believe that differences may have occurred in both groups and that colon surgery costs would represent a good approximation of overall high risk abdominal surgery costs. Finally, the cost-effectiveness analysis evaluated the scenario of one YLG, and there was no quality-adjusted life years (QALY) analysis, which is the recommended effectiveness measure in perioperative scenarios [32]. Considering the potential cost-savings associated with the bundle implementation, it is recommended to amplify the study to other centers and, if possible, follow patients after discharge to evaluate QALY in addition to the YLG.

## Conclusions

Our results indicate that implementing a high-risk surgical bundle may help improve the efficiency of healthcare delivery, potentially representing a valuable and cost-effective strategy for building a more sustainable healthcare system. The significant potential improvement in outcomes, such as reduction in one-year mortality indicated by this study, justifies the incremental costs of better caring for the most vulnerable postoperative patients. Micro-costing combined with cost-effectiveness analysis provided precise cost assessments for the redesigned bundle, demonstrating that this approach can improve efficiency using real-world financial data. These findings offer valuable insights for policymakers in developing sustainable patient-centered healthcare policies. Future research should focus on designing and testing improvement programs for high-risk surgical patients in diverse settings, considering the complexities of the healthcare system, identifying key processes for change, and refining economic evaluation methods.

## Supporting information

S1 File **S1 Table.** Median total cost and composition of costs in the two groups. **S1 Figure.** The graph demonstrates the impact on cost in the presence of the three most common complications in both groups. **S2 Figure.** Kaplan-Meyer analysis comparing survival with usual care vs. the new high-risk bundle. **S2 Table.** Statistical analysis of costs between groups. **S3 Table.** Cox multivariate regression model. **S3 Figure.** Decision to tree in the presence of postoperative infection, its impact on costs and increase in ICER. **S4 Figure.** Tornado diagram and univariate sensitivity analysis. **S5 Figure.** Tornado diagram and univariate sensitivity analysis – infection scenario.
(DOCX)

S2 File Major abdominal surgery bank. (XLSX)

S3 File Micro-costing – bank. (XLSX)
