# Supplementary material for: Cost-effectiveness analysis of a postoperative 48-hour care bundle for high-risk patients undergoing abdominal surgery
Source: PLoS One. 2025 Jun 3;20(6):e0320968. doi: 10.1371/journal.pone.0320968 (PMC12132966; doi:10.1371/journal.pone.0320968)
Supplement: S1 File — S1 Table. Median total cost and composition of costs in the two groups. S1 Figure. The graph demonstrates the impact on cost in the presence of the three most common complications in both groups. S2 Figure. Kaplan-Meyer analysis comparing survival with usual care vs. the new high-risk bundle. S2 Table. Statistical analysis of costs between groups. S3 Table. Cox multivariate regression model. S3 Figure. Decision to tree in the presence of postoperative infection, its impact on costs and increase in ICER. S4 Figure. Tornado diagram and univariate sensitivity analysis. S5 Figure. Tornado diagram and univariate sensitivity analysis – infection scenario. (DOCX) [file pone.0320968.s001.docx]

**Cost-Effectiveness Analysis of a Postoperative 48-Hour Care Bundle for High-Risk Patients Undergoing Abdominal Surgery**

**Supplementary Material**

**Table of contents**

S-Table 1. Median total cost and composition of costs in the two groups.

S-Fig 1. Impact on cost in the presence of the three most common complications in the new high-risk bundle or usual care.

S-Fig 2. Kaplan-Meyer analysis comparing survival with usual care vs. the new high-risk bundle.

S-Table 2. Statistical analysis of costs between groups.

S-Table 3. Cox multivariate regression model.

S-Fig 3. Decision to tree in the presence of postoperative infection, its impact on costs and increase in ICER.

S-Fig 4. Tornado diagram and univariate sensitivity analysis – infection scenario.

S- Box 1. Procedures included as major abdominal surgeries

**S-Table 1.** Median total cost and composition of costs in the two groups in the new bundle and usual care

| Cost $ | **New high-risk surgical bundle** | | | **Usual care** | | |
| --- | --- | --- | --- | --- | --- | --- |
|  | **All patients** | **Infection** | | **All patients** | **Infection** | |
|  | *n*=42 | YES=15 patients (34%) | NO=27 patients  (64%) | *n*=45 | YES=18 patients  (40%) | NO=27 patients  (60%) |
|  | Median  (1^st^/3^rd^ quartile) | Median  (1^st^/3^rd^ quartile) | Median  (1^st^/3^rd^ quartile) | Median  (1^st^/3^rd^ quartile) | Median  (1^st^/3^rd^ quartile) | Median  (1^st^/3^rd^ quartile) |
| Total | 1183.61  (676,52/ 1672.18) | 1825.38  (1201,73/ 3072.87) | 919.60  (503.20/ 1280.85) | 1033.49  (730.48/ 1526.88) | 1486.61  (1257.61/ 2034.04) | 833.43  (433.43/ 1071.02) |
| Medication | 169.58  (52.34/ 522.25) | 238.90  (123.29/642.36) | 158.99  (36.42/ 498.88) | 81.87  (35.22/ 242.75) | 169.58  (85.34/ 363.13) | 51.07  (23.08/ 140.04) |
| Structure | 322.29  (212.32/ 550.72) | 551.61  (442.32/ 1072.42) | 220.01  (194.55/ 325.43) | 312.24  (229.32/ 595.94) | 595.37  (372.82/ 806.66) | 238.65  (203.90/ 332.07) |
| Exams | 140.12  (73.53/ 271.66) | 203.78  (115.16/ 347.97) | 129.16  (52.67/225.30) | 165.85  (60.13/ 363.79) | 199.51  (131.44/ 395.43) | 153.44  (26.70/ 234.34) |
| Other professionals  Direct contact | 60.79  (42.19/ 97.80) | 106.67  (68.58/132.16) | 48.98  (38.38/ 66.29) | 72.84  (48.89/ 114.93) | 98.71  (70.41/ 145.49) | 57.04  (39.36/ 87.77) |
| Other professionals  Indirect contact | 100.20  (76.86/ 161.93) | 167.94  (117.44/ 216.42) | 85.30  (66.89/ 107.36) | 113.46  (78.99/ 170.63) | 164.16  (109.36/ 253.00) | 88.76  (64.95/ 124.49) |
| Medical  Direct contact | 30.79  (19.40/ 62.47) | 64.13  (31.84/ 98.12) | 21.35  (17.49/ 43.55) | 19.34  (15.36/ 45.31) | 49.59  (23.89/ 89.73) | 16.20  (12.14/ 19.73) |
| Medical  Indirect contact | 38.95  (25.83/ 62.52) | 62.79  (37.85/ 158.64) | 31.59  (22.82/ 44.48) | 20.84  (14.87/ 42.90) | 42.04  (21.35/ 117.38) | 16.06  (13.51/ 22.78) |

$ = US dollar; SD = standard deviation.

**S-Figure 1.** Impact on cost in the presence of the three most common complications in both groups. Blue represents the new high-risk bundle patient group and light orange the usual care group.


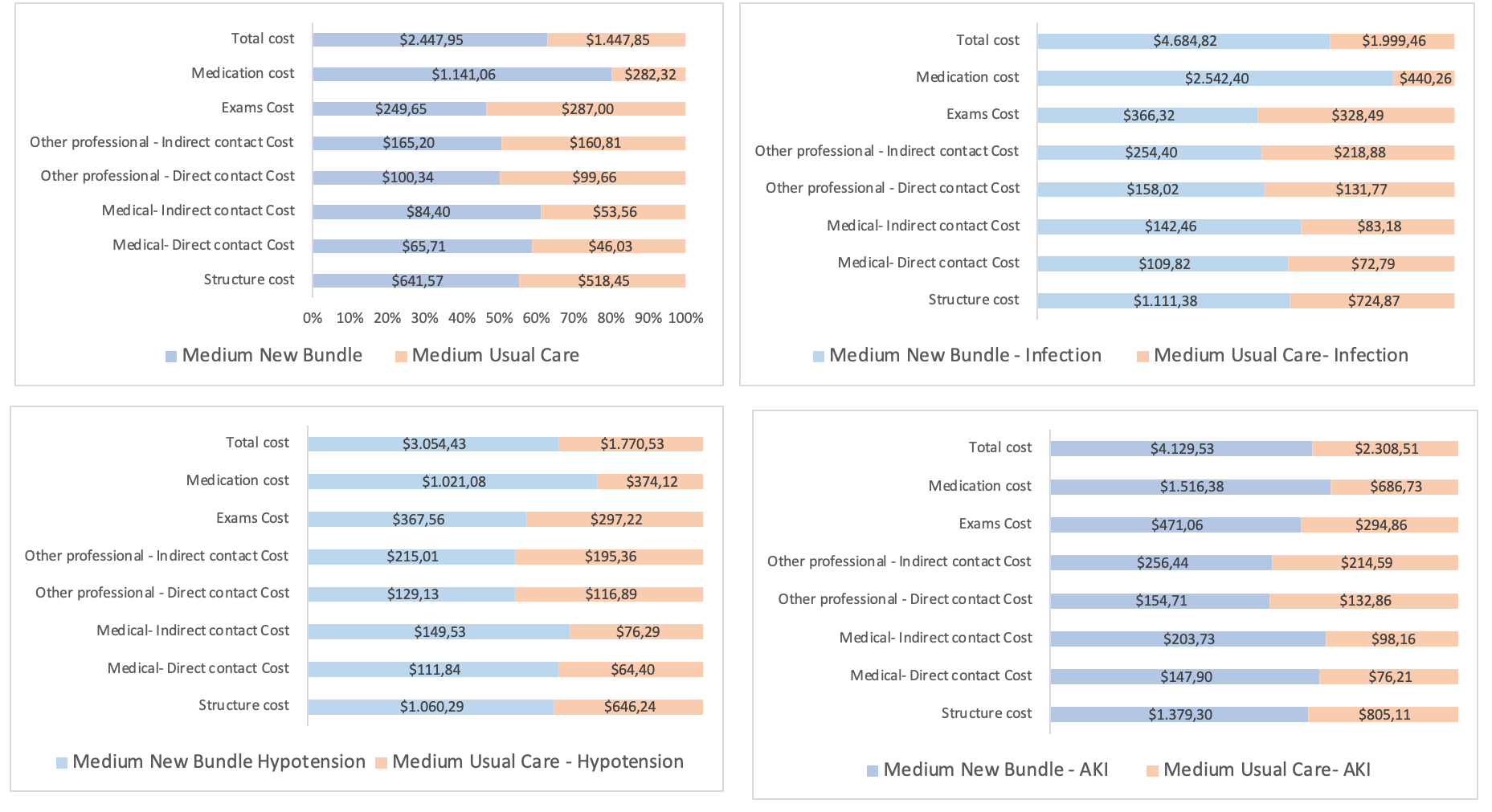


**S-Figure 2.** Kaplan-Meyer analysis comparing survival with usual care (red) vs. the new high-risk bundle (blue).


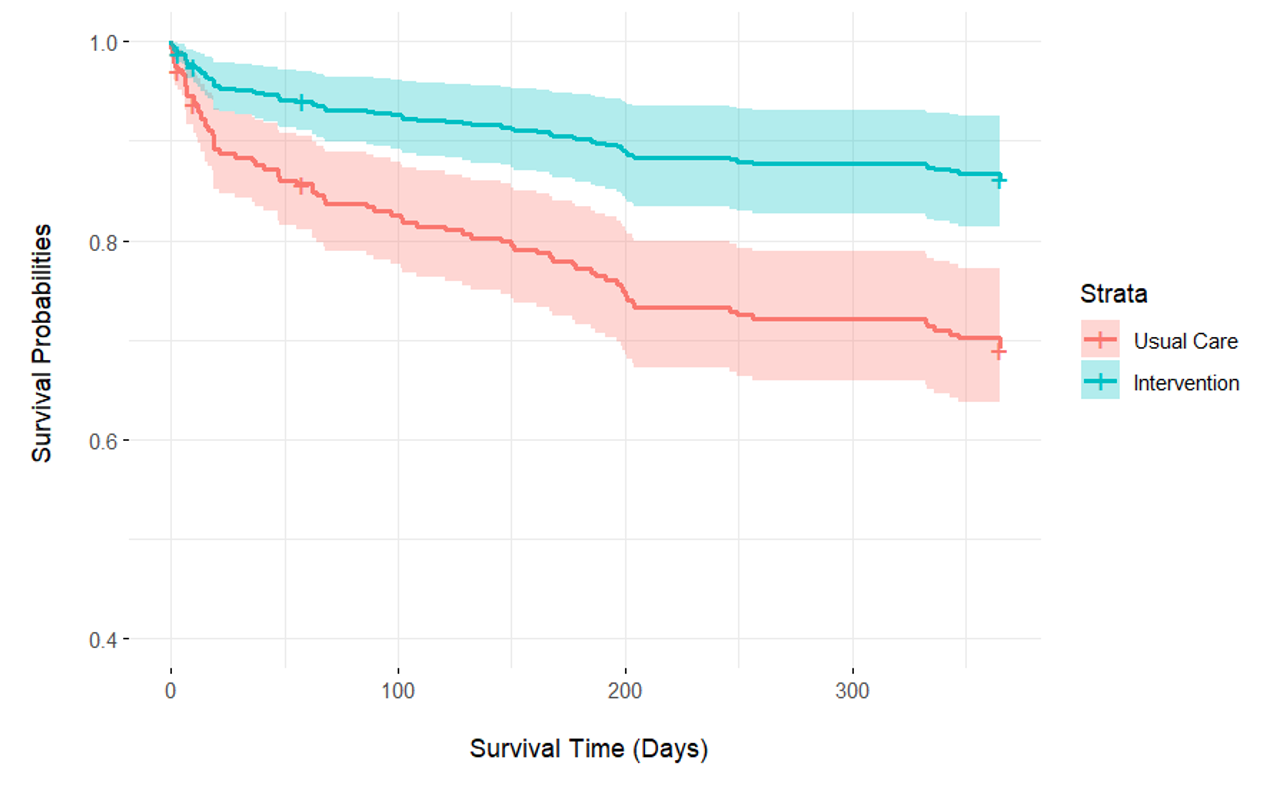


**S-Table 2.** Statistical analysis of costs between the new bundle and usual care

| **Cost** $ |  |  |  |  |  |  |
| --- | --- | --- | --- | --- | --- | --- |
| **Intervention** | **Infection** | **N** | **Median** | 1st Quartile | 3rd Quartile | **P** |
| **New High-Risk Surgical Bundle** | **-** |  | 1183.61 | 676.52 | 1672.18 | **0.3766*** |
|  | Yes | 15 | 1825.38 | 1201.73 | 3072.87 | <0.0005** |
|  | No | 27 | 919.60 | 503.20 | 1280.85 |  |
| **Usual Control** | **-** |  | 1033.49 | 730.48 | 1526.88 |  |
|  | Yes | 18 | 1486.61 | 1257.61 | 2034.04 | 0.0003** |
|  | No | 27 | 833.43 | 433.43 | 1071.02 |  |

p-value for Mann-Whitney-Wilcoxon Test comparing groups. **p-value for Mann-Whitney-Wilcoxon Test comparing infection status within group. $ Dollar.

**S-Table 3.** Cox multivariate regression model for one-year survival

|  | HR | 95% CI | p-value |
| --- | --- | --- | --- |
| High-risk bundle | 0.40 | 0.24–0.66 | 0.0003 |
| EXCARE > 10% | 2.29 | 1.42–3.67 | 0.0005 |
| Infection | 1.86 | 1.17–2.93 | 0.007 |

HR = hazard ratio; CI = confidence interval.

**S-Figure 3.** Decision tree in the presence of postoperative infection, its impact on costs and increase in ICER. $ = US dollar; LY = life year; LYG = life year gained; ICER = incremental cost-effectiveness ratio.


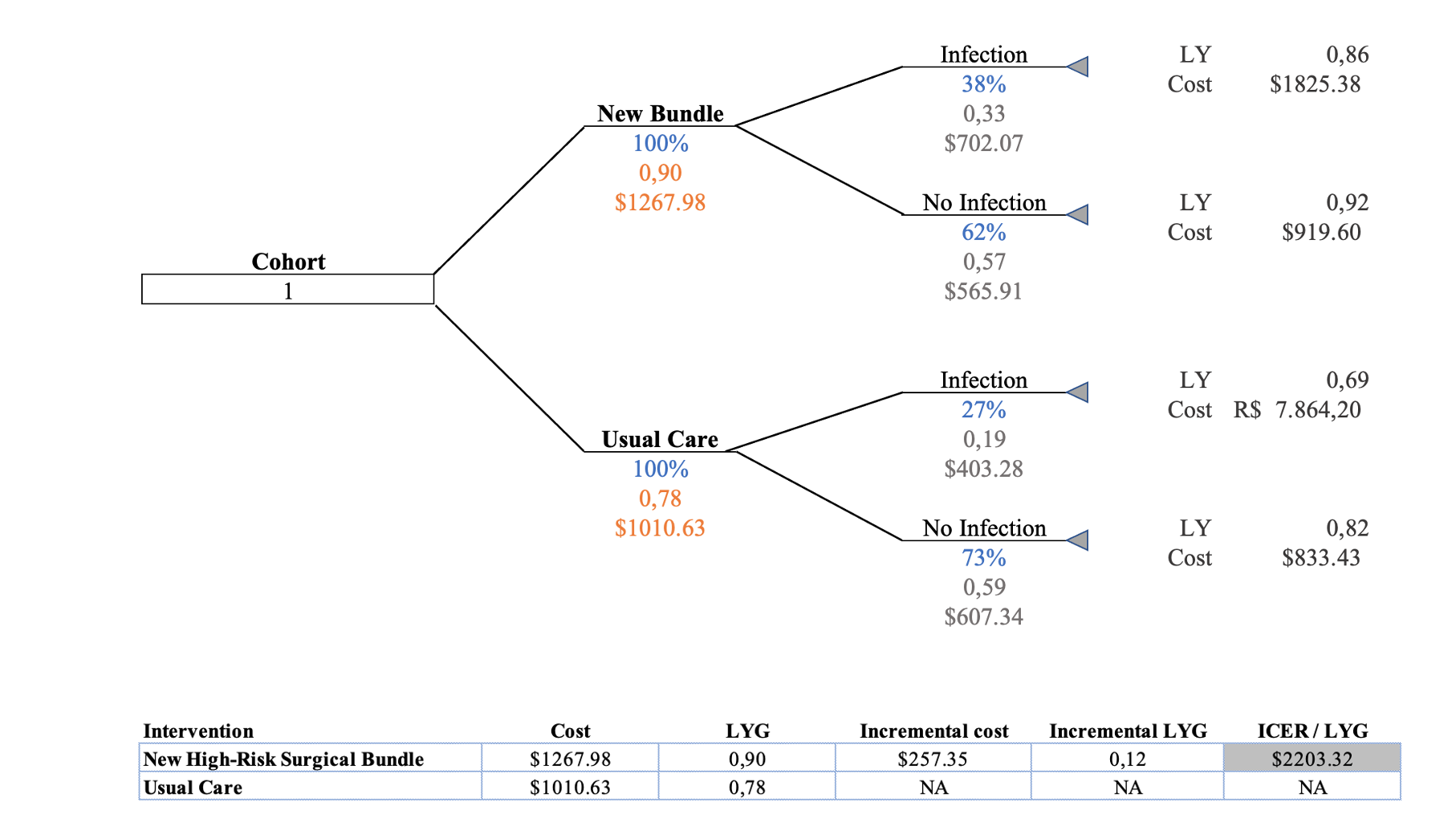


**Cost of patients with complications**

Index complications were identified as the first markers of clinical deterioration. The most prevalent complications were hypotension present in 40% of the patients in the high-risk bundle group and in 44% in the usual care group. The cost of patients with hypotension increased by 25%. Infection was present in 36% (15/42) of patients in the new care bundle, resulting in the greatest difference in the mean cost with an increase of 91%. Infection was also common in the usual care (40% – 18/45), which generated an average increase in total cost of 38%. Acute kidney injury (AKI) occurred in 26% (11/42) of the high-risk bundle group and in 20% (9/45) in the usual care group, which increased the average total cost by 68% and 59%, respectively.

**S-Figure 4.** Tornado diagram and univariate sensitivity analysis. $ Dollar.


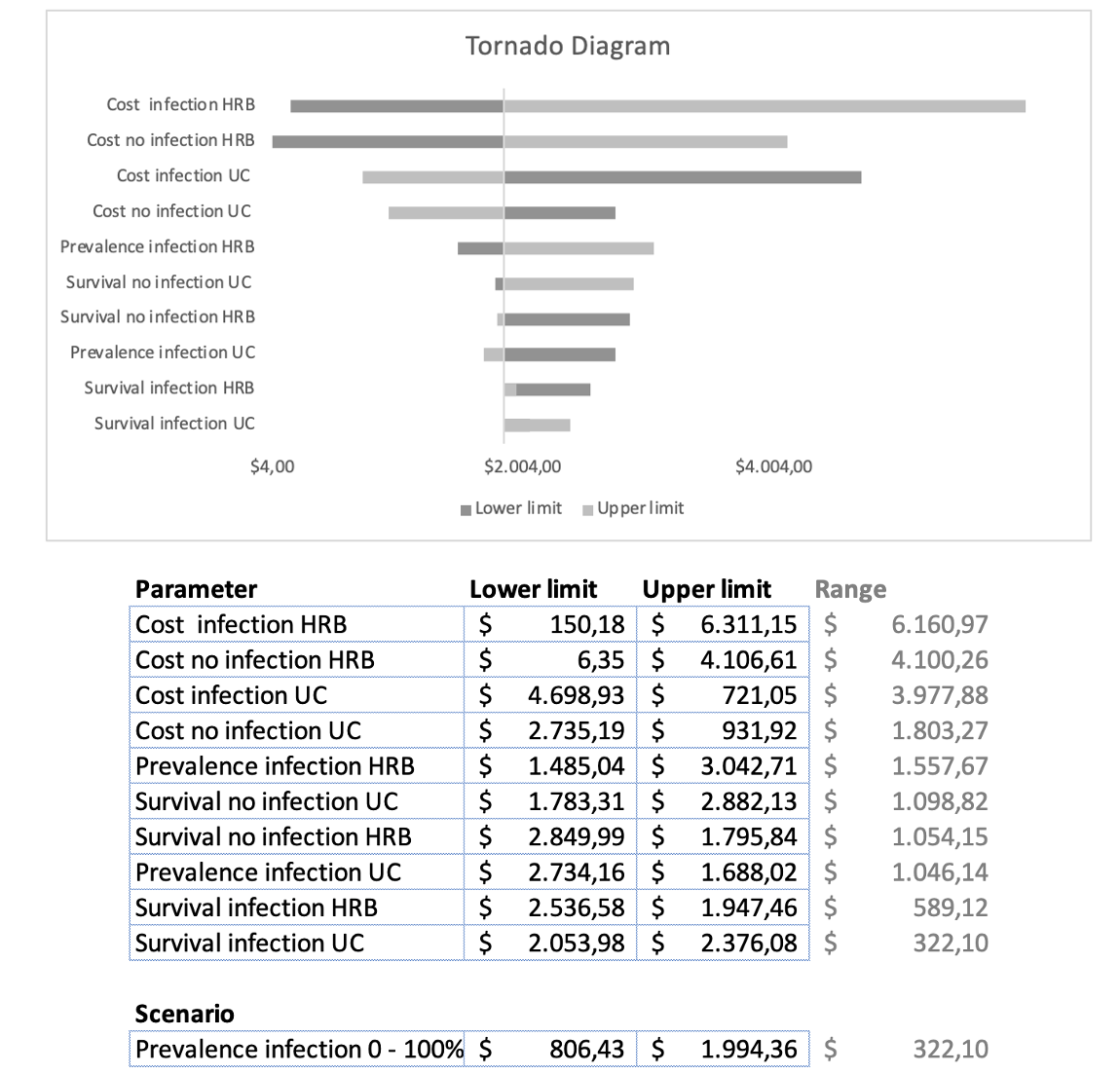


**S Box 1 – Procedures included as major abdominal surgeries**

| **Surgical Procedure** | **Usual Care** | **High-risk Bundle** |
| --- | --- | --- |
| Adrenalectomy | 0 | 1 |
| Colon lowering | 1 | 0 |
| Abdominoperineal rectal amputation | 6 | 6 |
| Cystectomy | 5 | 5 |
| Colectomy, hemicolectomy/retosigmoidectomy | 45 | 42 |
| Biliodigestive diversion | 0 | 1 |
| Enterectomy/enteroenteroanastomosis | 6 | 1 |
| Esophagectomy | 1 | 2 |
| Resection of retroperitoneal tumor | 0 | 3 |
| Exploration of biliary tract | 5 | 3 |
| Gastrectomy | 8 | 8 |
| Gastroenteroanastomosis | 1 | 6 |
| Gastroplasty | 7 | 10 |
| Hepatectomy | 3 | 3 |
| Iliotransversoanastomosis | 1 | 0 |
| Exploratory laparotomy | 75 | 35 |
| Retroperitoneal lymphadenectomy | 6 | 1 |
| Nephrectomy | 13 | 3 |
| Pancreatectomy | 1 | 0 |
| Ulcerorrhaphy | 4 | 0 |
| **Total** | **188** | **130** |
